# Oncologists’ Perspectives on Ketogenic Diets in Pediatric Brain Cancer: Potential, Challenges, and the Path Forward

**DOI:** 10.3390/nu17172843

**Published:** 2025-08-31

**Authors:** Hanan AlMutairi, Madhumita Dandapani, Khawar Siddiqui, Fiona McCullough

**Affiliations:** 1 Clinical Nutrition Department, King Faisal Specialist Hospital and Research Center, Riyadh 13324, Saudi Arabia; 2 Children’s Brain Tumor Research Center, Biodiscovery Institute, University of Nottingham, Nottingham NG7 2RD, UK; madhumita.dandapani@nottingham.ac.uk; 3 Department of Pediatric Hematology/Oncology, King Faisal Specialist Hospital and Research Center, Riyadh 13324, Saudi Arabia; ksiddiqui@kfshrc.edu.sa; 4 Department of Bioscience, University of Nottingham, Nottingham NG7 2RD, UK; fiona.mccullough@nottingham.ac.uk

**Keywords:** ketogenic diet, pediatric oncology, brain neoplasms, diet therapy, healthcare surveys, Saudi Arabia

## Abstract

**Background/objectives**: Treating pediatric brain tumors remains challenging due to the limitations of conventional therapies, which often damage healthy cells. Ketogenic diets (KDs)—high in fat and low in carbohydrates—have emerged as potential adjunct therapies by limiting glucose availability and offering ketones as an alternative energy source proposed to hinder tumor growth. However, due to limited awareness, there is hesitancy to recommend KDs. **Methods**: This study assessed oncologists’ knowledge and perceptions in Saudi Arabia regarding KD use in pediatric brain cancer patients. A cross-sectional survey was conducted with 94 oncologists from five major Riyadh healthcare centers, examining their knowledge, safety concerns, feasibility, and perceived efficacy of KDs. **Results**: Results showed that 67% correctly identified the basic composition of KDs, though 43% were neutral about its safety. Concerns about malnutrition and side effects were common and 53% found adherence to the diet challenging in pediatric patients. While 48.9% believed KDs could improve outcomes when combined with standard therapies, many stressed the importance of tailoring recommendations to individual medical conditions, including patient age, treatment stage, and overall nutritional status. Participants’ responses revealed variability in knowledge and perception levels regarding KDs, with consultants and internationally educated oncologists generally expressing more favorable views compared to fellows, who showed greater hesitancy. **Conclusions**: This study shows a mixed level of knowledge and perceptions among oncologists, reflecting a lack of consensus about KDs’ safety, feasibility, and potential benefits. These findings suggest the need for further education, clearer guidelines, and interdisciplinary collaboration to support informed decision-making, particularly in the local context.

## 1. Introduction

Despite ongoing improvements in screening methods, early detection, and therapeutic interventions, cancer continues to remain one of the most formidable health challenges [1,2]. Cancer is the second leading cause of death worldwide, following cardiovascular disease. Malignant brain tumors remain a significant health concern, causing significant morbidity and mortality among adults, while also ranking as the leading cause of cancer-related fatalities in children [3,4,5,6]. Globally, the incidence of pediatric brain tumors is estimated at approximately 3–6 cases per 100,000 children each year, making them the most common solid tumor in childhood [7].

Existing treatments for malignant brain tumors often fall short in offering sustainable solutions, as they primarily target the abnormalities within tumor cells, potentially compromising the well-being and functionality of healthy brain cells [8,9,10]. This challenge has prompted interest in alternative approaches, including dietary interventions such as ketogenic diets (KDs). The ketogenic diet is characterized by a high-fat, very low-carbohydrate, and moderate-protein composition. In classic therapeutic forms—commonly used in pediatric epilepsy—the fat-to-(carbohydrate + protein) ratio is typically around 4:1, which translates to approximately 70–80% of total calories from fat, 10–15% from protein, and 5% or less from carbohydrates [11]. For some modified protocols, including those studied in brain tumor contexts, carbohydrate intake may be restricted to around 20 g/day (~3–5% of total energy), with fat comprising approximately 70% and protein around 20%. Depending on a child’s age, activity level, weight, and clinical goals, total daily caloric intake may range from 20 to 80 kcal/kg, though individualized planning by a registered pediatric dietitian is essential [12].

Despite public curiosity, its application in pediatric oncology lacks robust scientific validation. There is currently limited evidence of positive findings from well-designed randomized controlled trials (RCTs) or official guidelines supporting KD use in childhood cancer treatment. It has been previously reported in preclinical models that diet restriction exerts anti-inflammatory and pro-apoptotic effects in human malignant gliomas [13]. A KD aims to reduce circulating glucose and glycolysis, thereby targeting tumor metabolic vulnerabilities [10,14,15].

Experimental studies suggest KDs may elevate oxidative stress by shifting tumor metabolism towards mitochondrial respiration, increasing reactive oxygen species (ROS) and promoting tumor cell apoptosis. KDs may also modulate pathways such as PI3K/Akt/mTOR and activate AMPK signaling, thereby inhibiting tumor growth [10,11]. However, robust evidence supporting KDs in pediatric oncology is limited.

Healthcare professionals (HCPs), particularly physicians, play a key role in guiding families through complex treatment decisions, including the consideration of dietary interventions such as the ketogenic diet (KD) in pediatric brain cancer care. While the ketogenic diet (KD) has been proposed as promising adjunctive treatment for pediatric brain cancer due to its potential to exploit tumor metabolic vulnerabilities, its implementation is not without challenges. One of the primary concerns is the potential for side effects, which may include hypoglycemia, gastrointestinal discomfort, constipation, dehydration, kidney stones, and growth retardation, particularly in long-term use [16,17]. Ultimately, many physicians remain hesitant to recommend KDs [18,19].

In young children undergoing intensive cancer treatment, these complications may exacerbate already fragile nutritional and metabolic states. Safety and compliance also pose significant hurdles, as maintaining strict macronutrient ratios requires intensive dietary planning, constant monitoring, and precise preparation of meals—often during a time when families are already overwhelmed emotionally and logistically by the child’s illness. The psychosocial burden on families is considerable, as parents must not only manage complex medical regimens but also enforce restrictive eating patterns, often in hospital settings with limited dietary support. These factors contribute to the reluctance among healthcare professionals to recommend or initiate the ketogenic diet in oncology care. Many clinicians cite a lack of standardized clinical protocols, insufficient training in dietary therapies, and concerns about interference with standard treatments or overall nutritional adequacy [20,21]. As a result, despite encouraging preliminary evidence, the ketogenic diet remains a potentially underexplored strategy in pediatric neuro-oncology, warranting further research, interdisciplinary collaboration, and structured clinical guidelines to ensure its safe and effective integration.

To address the limited clinical data on KDs in pediatric brain tumors, our team is currently conducting a clinical trial evaluating the diet’s feasibility and therapeutic potential in this patient population. However, during the recruitment phase, we encountered notable hesitation from several pediatric oncologists who expressed concerns about the safety and practicality of administering KDs to their patients. This reluctance highlighted an urgent need to understand clinicians’ knowledge, perceptions, and potential barriers to KD implementation. As a result, we developed this survey to explore these perspectives and inform future integration efforts.

Understanding oncologists’ knowledge and perceptions of KDs is vital for developing evidence-based guidelines and effective communication strategies. Therefore, this study aimed to explore medical oncologists’ knowledge, perceptions, and attitudes towards KDs in pediatric brain cancer in Saudi Arabia. By identifying gaps and concerns, this research can guide future education and interdisciplinary collaboration among dietitians, oncologists, and researchers to inform safe and effective use of KDs as a potential adjunct therapy. To our knowledge, this is the first survey in Saudi Arabia and only the second worldwide focused specifically on KD use in pediatric brain cancer, providing region-specific insights into clinical attitudes and barriers to implementation.

Clinicians’ knowledge and perceptions are critical factors influencing whether emerging or supportive therapies, such as the ketogenic diet, are discussed or integrated into care plans. Understanding these perspectives can guide the development of educational strategies and clinical protocols that align with practitioner concerns. While the KD has gained attention as a potential adjunct in brain tumor management, it remains largely absent from established pediatric oncology guidelines, with limited formal endorsement due to insufficient large-scale clinical evidence. This gap underscores the importance of exploring physician attitudes to inform future research and policy development.

## 2. Materials and Methods

### 2.1. Study Design

A total of 94 oncologists participated in this cross-sectional survey, which targeted consultants, assistant consultants, fellows, and residents working in five major oncology centers in Riyadh, Saudi Arabia. Participants received the questionnaire in an online fashion using convenience sampling techniques via a QR code or URL. Prior to the commencement of the survey, all participants were provided an introductory statement. This statement clearly explained the study’s purpose, the estimated time to complete the survey, and how to contact the researchers. Survey was open for 3 months from November 2023 till February 2024. Participation was voluntary and anonymous, with no incentives offered. Ethical approval for the study was granted by the University of Nottingham under approval number 202611832/10378165, ensuring adherence to the ethical principles outlined in the Declaration of Helsinki and its subsequent amendments.

Eligible participants were oncologists, including consultants, assistant consultants, fellows, and residents working in the selected hospitals. There were no additional exclusion criteria beyond professional role and willingness to participate. Participants were asked to provide demographic information, including their designation, gender, years of experience, and medical education background. They were then presented with questions related to their knowledge of the ketogenic diet, including its composition, safety and feasibility profile, and potential benefits in oncology. Additionally, oncologists were asked about their willingness to recommend the KD to cancer patients, their concerns about its implementation, and the reasons behind not using the KD in oncology practice.

To assess the foundational knowledge of KDs among participants, we included two preliminary questions in the survey related to the basic principles of the ketogenic diet—specifically its macronutrient composition and whether it follows a uniform regimen. Based on responses to these two questions (Q5 and Q6), participants were categorized into two groups: “perception-positive”, indicating correct answers to both questions and thus a basic familiarity with KDs, and “perception-negative”, indicating incorrect answers or uncertainty. This grouping was not intended to reflect comprehensive knowledge or clinical expertise but rather to differentiate between participants with or without preliminary awareness.

### 2.2. Survey Development

The survey questions were developed as a two-step process by the Principal Investigator (PI), a pediatric oncology dietitian drawn with extensive clinical expertise, from both University of Nottingham in the UK and the King Faisal Specialist Hospital in Saudi Arabia. An initial version was piloted with a small group of medical students (*n* = 17) to assess clarity, comprehensibility, and relevance. Feedback from this pilot was used to refine question wording and structure, resulting in the final 18-item questionnaire. No formal psychometric validation was conducted. We recognize this as a key limitation, as it may affect the reliability and consistency of the survey outcomes. Future studies should consider validating survey instruments through standard psychometric procedures.

These questions addressed common knowledge and concerns among medical oncologists and included questions related to ketogenic dietary approaches in pediatric cancer patients. Questionnaires about KDs in other medical conditions including obesity and diabetes were also taken into consideration while developing this survey. The initial survey contained 16 multiple choice questions and was conducted in Riyadh with medical students. The results from the data obtained were analyzed and questions were modified to avoid any confusion and refine the focus of the survey.

The final survey consisted of three sections:

- Section A (Q1–Q4) collected demographic data, including designation, gender, years of experience, and medical education background.
- Section B (Q5–Q11) assessed basic knowledge and awareness of the KD, including its composition and general principles.
- Section C (Q12–Q18) explored perceptions of the KD’s safety, feasibility, and effectiveness, along with concerns about its use in pediatric oncology.

The full questionnaire is provided in Appendix A.

### 2.3. Data Management and Statistical Analysis

The survey was administered using Jisc Online Surveys, a secure, GDPR-compliant web-based platform designed for academic research and widely used across UK higher education institutions (https://app.onlinesurveys.jisc.ac.uk/, accessed on 1 November 2023). We administered the survey in a format for anonymity, convenience for participants, and for straightforward, accurate analysis of the collected data by providing the QR code or URL in-person to the participant.

Inferential statistics were applied to explore associations between categorical demographic variables and survey responses. Chi-square tests or Fisher’s exact tests (where appropriate) were employed to determine the statistical significance of these associations. The level of significance was set at *p* < 0.05 for all statistical tests.

To further explore complex relationships among multiple categorical variables, multiple correspondence analysis (MCA) was conducted using the Factoshiny package in R (version 4.0.5; “Shake and Throw”, The R Foundation for Statistical Computing). MCA extends correspondence analysis to explore associations among several categorical variables, as seen in survey datasets. It translates both respondents and response categories into a shared low-dimensional space, typically two or three axes, where proximity signals stronger associations. Categories that co-occur frequently cluster together and respondents with similar response patterns appear nearby. This graphical mapping reveals latent structures, respondent segments, and meaningful relationships that would remain hidden in simpler bivariate analyses. MCA’s simultaneous, multivariable perspective provides a nuanced, integrative view of complex categorical data. The first two dimensions (Dim.1 and Dim.2), representing the categories contributing most significantly to the overall variance, were used to construct bi-plots. In these plots, the proximity between row or column points reflects the similarity of their profiles. The variable plots illustrated the variables most strongly associated with each dimension, based on their squared correlations.

Due to the categorical structure of the survey responses, inferential analyses were conducted using chi-square or Fisher’s exact tests and MCA was employed to explore multidimensional associations. Multivariate regression analysis was not applied, as the data did not include continuous dependent variables or sufficient predictors to justify a more complex model.

All statistical analyses were performed using IBM SPSS Statistics for Windows; Version 20.0 (IBM Corp.; Armonk, NY, USA) and R statistical software.

## 3. Results

### 3.1. Demographic Characteristics of Participiants

A total of 94 oncologists participated in the survey (73% response rate), with a balanced gender distribution and a wide range of experience levels. Detailed demographic characteristics are presented in Table 1.

These educational backgrounds reflect the diversity in exposure to global and local clinical practices, potentially influencing clinical decision-making and familiarity with emerging therapies such as dietary interventions. Importantly, statistical analysis revealed no significant differences in demographic characteristics, including gender, professional role, clinical experience, or source of medical education, between perception-positive and perception-negative groups. This suggests that factors other than demographic background—such as clinical exposure, institutional support, or personal interest in integrative therapies—may influence attitudes toward KDs in pediatric oncology care.

### 3.2. KD Knowledge

Regarding knowledge of the ketogenic diet, exactly half (47/94) of oncologists correctly identified it as being high in fat and low in carbohydrates, highlighting a notable knowledge gap about its basic composition among half of the surveyed participants. Sixty-nine (73.4%) of respondents did not agree with the fact that KD is a restricted diet with a uniform regimen for everyone; however, a smaller group of respondents (26.6%) agreed with this statement. Regarding the source of first even exposure to the terminology of the KD, 34 (36.2%) responded that they heard about it from social media platforms.

### 3.3. Concerns About KDs

Participants showed a wide range of opinions regarding the safety and feasibility of KDs, with many selecting neutral responses and relatively few considering it very safe. Common concerns included risk of malnutrition and challenges with adherence. Detailed response distributions have been presented in Table 2.

To assess the perception of participants about KDs, the questionnaire was divided into three categories:

- Section 1: KD safety (includes Q8, 12, 13, and 16).
- Section 2: KD feasibility (includes Q9 and 14).
- Section 3: KD effectiveness (includes (Q11, 17, and 18).

Based on the results from Q5 and Q6, participants were classified into two categories: Category A, for those with basic information (perception-positive group, *n* = 47, 50.0%), and Category B, for those without basic KD information (perception-negative group, *n* = 47, 50.0%). This classification was based on foundational knowledge of the KD, such as its composition (high fat, low carbohydrate) and general dietary principles. It is important to note that these labels do not reflect adherence to clinical guidelines but were used as working categories to differentiate participants based on their basic level of awareness as captured through the survey. The responses to the questions described in the three sections stated above were analyzed to determine the participants’ awareness of KDs by the perception groups. Association of these expressions was analyzed with their demographic and supplementary data using univariate and multivariable analysis.

### 3.4. Perception Analysis

The statistical analysis of the perception-positive group, composed of individuals knowledgeable and aware of the ketogenic diet (KD), there were notable demographic trends. The group was primarily composed of consultants, followed by fellows. Gender distribution was relatively balanced and most participants had 20 or fewer years of professional experience. In terms of educational background, the majority were educated nationally, with a smaller proportion having received international training. These descriptive statistics provide a detailed overview of the demographic characteristics of participants with a positive perception of the ketogenic diet; however, no statistically significant differences were observed between this group and those with negative perceptions across variables such as gender, clinical role, years of experience, or source of medical education. (Table 1).

### 3.5. Perception-Positive Group

#### 3.5.1. Safety (Q8, 12, 13, and 16)

Perception-positive participants expressed varied views on KD safety, with many indicating neutral or cautious opinions. Specific response distributions are shown in Table 2. Finally, regarding, a wide spectrum of one or more concerns about administering KDs to pediatric cancer patients were recorded. The major concern raised by the participants was the “risk of weight loss or malnutrition” as well as “risk of unknown medical side effects” followed by “lack of supervision from nutritional services” (Table 3).

#### 3.5.2. Feasibility (Q9 and Q14)

In relation to the ease of following the KD in pediatric cancer patients, the majority of perception-positive participants found KDs difficult to implement in pediatric patients and preferred its use in those off active treatment (refer to Table 3 for response breakdowns.).

#### 3.5.3. Effectiveness (Q11 and Q17)

In relation to effectiveness, more than half of the perception-positive participants (55.3%, *n* = 26) were of the view that the KD is ineffective in improving treatment outcomes in oncology along with standard of care. However, there was a divided opinion on recommending KDs to the patient following a request from a patient or their parents. Participant responses were divided regarding whether they would approve the administration of KDs upon patient request, with some supporting it, some opposing it, and others expressing a generally positive attitude (Table 2 and Table 3).

### 3.6. Perception-Negative Group

Statistical analysis of respondents with a negative perception of the ketogenic diet (KD)—particularly those identified as needing improved awareness—suggested emerging trends in relation to specific demographic variables, although these were not statistically significant.

Fellows made up the largest subgroup in the perception-negative group. Many participants expressed uncertainty about the KD’s safety, feasibility, and use. Notably, the most common concern was the risk of malnutrition (Table 2).

#### 3.6.1. Safety (Q8, 12, 13, and 16)

Regarding the safety of administering a ketogenic diet (KD) to pediatric cancer patients, a significant proportion of participants (*n* = 23 (48.9%)) maintained a neutral stance. In contrast, several participants expressed concerns about the potential risks associated with KDs. Among the perception-negative participants, almost half indicated an equivocal response regarding willingness to allow oncology patients to follow a KD. When it came to concerns about recommending KDs to cancer patients, a substantial majority of perception-negative participants reported having one or more reservations. The primary concern highlighted was the “risk of weight loss or malnutrition,” noted by several participants (Table 2 and Table 3).

#### 3.6.2. Feasibility (Q9 and Q14)

When asked about the ease with which pediatric cancer patients can follow a ketogenic diet (KD), most perception-negative participants believed it to be challenging, while very few considered it easy to adhere to. Preferences regarding the timing of KD use were mixed; some participants supported its use during active treatment such as radiotherapy or chemotherapy, whereas an equal number indicated they would not recommend KDs for any pediatric cancer patient (Table 3).

A notable proportion of respondents selected “neutral” for questions related to the KD’s safety, feasibility, and effectiveness. This pattern may reflect genuine uncertainty or insufficient knowledge about the KD’s clinical use in pediatric oncology, highlighting a lack of consensus. It may also indicate social desirability bias, where participants preferred to give a non-committal answer rather than admit unfamiliarity or take a definitive position. Similar patterns have been noted in surveys of physicians’ attitudes towards emerging or unconventional therapies [20,22]. These findings further emphasize the need for clear, evidence-based guidance and accessible training to reduce uncertainty and support oncologists in making informed recommendations.

#### 3.6.3. Effectiveness (Q11, 17, and 18)

Most perception-negative participants (54.3%, *n* = 26) believed that incorporating a ketogenic diet (KD) alongside standard care can improve treatment outcomes in oncology. However, opinions were divided regarding whether to recommend the KD upon request from patients or their parents. Some participants stated they would disapprove of administering KDs based on patient or parent requests, while others were in favor. A considerable portion remained uncertain, noting that their decision would depend on the patient’s specific medical condition (Table 3).

A substantial number of respondents selected “neutral” responses for questions related to the KD’s safety, feasibility, and effectiveness. This trend was observed in both perception-positive and perception-negative groups. While this may reflect genuine uncertainty or lack of familiarity with KDs in pediatric oncology, it could also suggest social desirability bias—where participants avoid strong statements on less familiar or controversial topics. Similar trends have been noted in physician surveys addressing novel or non-standard therapeutic approaches [20,22].

### 3.7. Results of Multiple Correspondence Analysis

**Figure 1:**
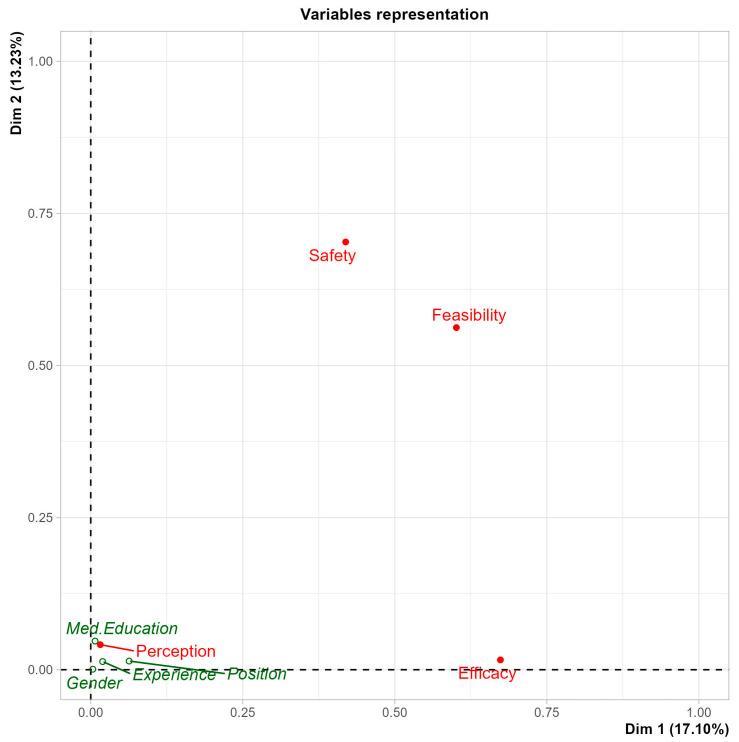
Multiple correspondence analysis bi-plot representation of variables of interest: the variable plots help to identify variables that are the most correlated with each dimension. The dimensions represent categories with the highest contribution and the highest fraction of the total variance in the data. X- and y-axes represent the first and second dimension (Dim.1 and Dim.2) of the MCA analysis performed on physicians’ perception towards the safety, feasibility, and efficacy.

**Figure 2:**
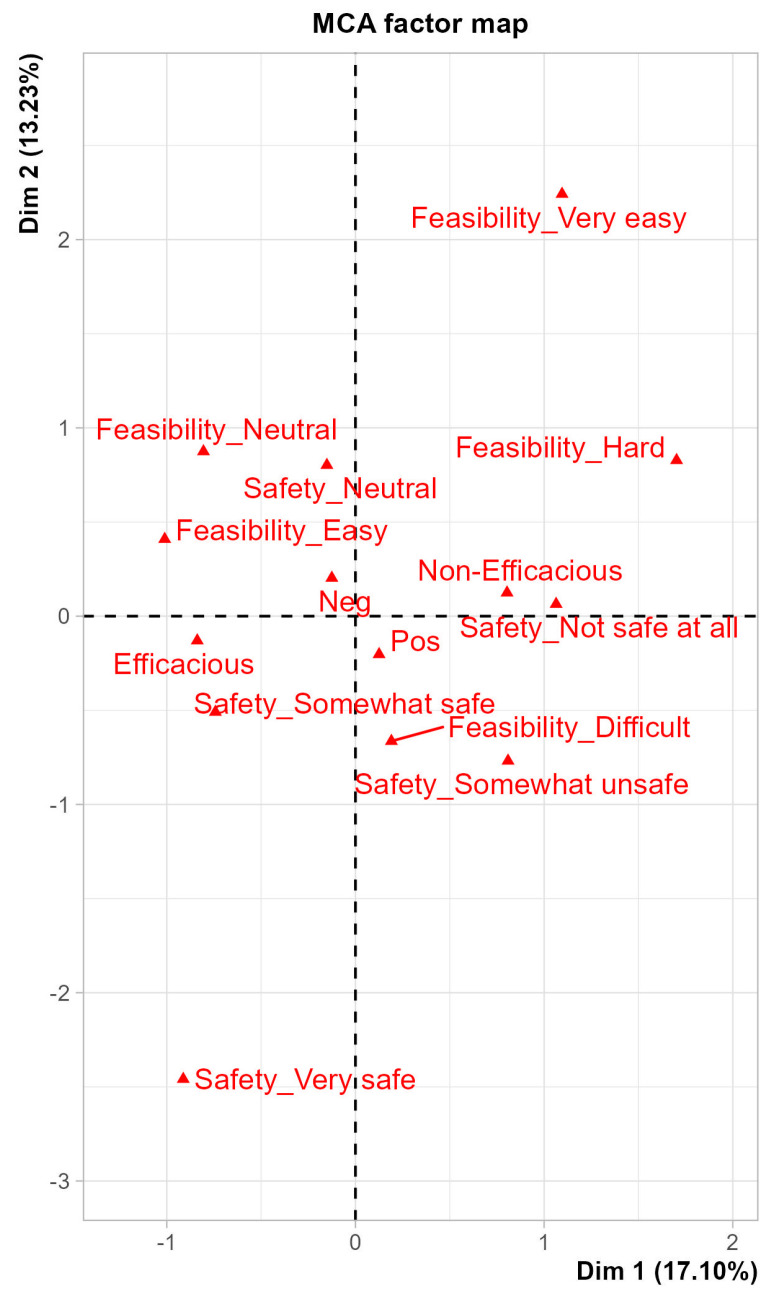
Multiple correspondence analysis—descriptive of factors: the distance between any row points or column points gives a measure of their similarity (or dissimilarity). Row points with similar profile are closed on the factor map. The same holds true for column points.

Multiple correspondence analysis (MCA) performed on physicians’ perception towards the safety, efficacy, and feasibility of KDs with their knowledge and perception of KDs. Gender (male or female), position (Consultant, Assistant Consultant, Fellow or Resident), professional experience in the field (Table 1) and source of medical education (national only, international or international and national) were taken as supplementary variables. MCA showed that perceptions of KD safety and feasibility were closely associated with perceptions of efficacy, suggesting these dimensions are interrelated in shaping clinicians’ overall attitudes. In contrast, these dimensions did not appear to cluster meaningfully with physicians’ self-reported knowledge of KDs (Supplementary Tables S1–S4, Figure 1 and Figure 2).

Dimension 1 (Dim.1) primarily represented the gradient from concern and uncertainty (e.g., neutral or negative responses regarding KD safety and feasibility) to greater perceived safety and openness to KD use. Dimension 2 (Dim.2) captured variation in professional role and willingness to recommend KDs. On the MCA biplot (Figure 1), variables closer together reflect similar response patterns across participants, while those located far apart represent contrasting profiles. For example, perception-positive oncologists tended to cluster near responses indicating potential KD safety and conditional approval, while perception-negative participants clustered closer to “neutral” or “unsure” responses.

These patterns provide a visual representation of how oncologists’ perceptions, roles, and training may influence their attitudes, although these findings remain exploratory.

These associations may reflect differences in training or exposure, but causality cannot be assumed. Further research is needed to explore whether perception-positive trends align with evidence-based clinical behaviors.

## 4. Discussion

This study is the first in Saudi Arabia to explore oncologists’ knowledge and perceptions of the ketogenic diet (KD) as a potential complementary approach for pediatric brain cancer. The findings offer initial insight into local oncologists’ understanding and perceptions of KDs and underscore both the opportunities and challenges of its future integration in Saudi pediatric oncology settings. Although preclinical studies and small feasibility trials have explored KDs in brain tumors, robust clinical evidence in pediatric oncology remains scarce [16,18].

The ketogenic diet’s proposed mechanism of action in brain tumors centers on exploiting the altered energy metabolism of cancer cells. Brain tumors often depend heavily on glucose and glycolysis for rapid growth, known as the Warburg effect [10]. By restricting carbohydrates and increasing fat intake, the KD shifts the body’s primary energy source to ketone bodies, which most tumor cells cannot efficiently utilize due to mitochondrial dysfunction [11]. This metabolic shift may increase oxidative stress within tumor cells through elevated mitochondrial ROS production, leading to apoptosis [15]. The KD has also been shown to influence signaling pathways such as downregulating the PI3K/Akt/mTOR pathway and activating AMPK, which can inhibit tumor cell proliferation and survival [13,19]. However, it is important to note that these mechanisms are largely supported by preclinical studies or early-phase clinical observations. Robust clinical trial evidence in pediatric oncology remains limited and further research is required to confirm these proposed effects in real-world settings [16,17,18]. Together, these effects provide a theoretical basis for using KDs as an adjunctive strategy to sensitize tumors to standard therapies while potentially protecting normal brain tissue. It is also important to acknowledge that the survey instrument, while informed by existing literature and pilot tested, was not subjected to formal psychometric validation. This may affect the reliability and internal consistency of the responses and represents a methodological limitation. Future iterations of the survey should incorporate psychometric testing methods such as internal consistency measures and construct validation to enhance reliability and interpretability of results.

The participation rate of 73% suggests notable interest among oncologists in dietary interventions in pediatric oncology. While Klassen et al. (2020) serves as a useful reference, it focused more broadly on oncologists’ views of KDs and low-sugar diets across various cancer types including CNS. To our knowledge, our study is the only one to date that specifically examines oncologists’ perceptions of KDs in pediatric brain cancer, making it the most directly comparable, albeit methodologically distinct, study [22]. Moreover, our study reveals that social media was the most common source of information about the KD for participants (36.2%). This may reflect the lack of structured, evidence-based education on this topic in formal medical training. This points to a clear need for targeted educational programs and reliable resources to help oncologists understand the principles, mechanisms, and appropriate use of KDs. This limited awareness of the fundamental principles of the ketogenic diet indicates an important knowledge gap among oncologists. As accurate understanding of the KD’s composition is foundational for evaluating its potential risks, benefits, and practical implementation, this gap underscores the need for targeted education and training to ensure oncologists can provide accurate information to patients and families considering dietary interventions.

Interestingly, while only about half of respondents agreed that the KD could improve treatment outcomes, nearly three-quarters indicated they would be willing to recommend it. This apparent contradiction may reflect a tendency among physicians to acknowledge caregiver preferences or remain cautiously open to complementary approaches when requested, even in the absence of robust evidence. This pattern has been suggested in studies examining physician attitudes toward integrative or non-standard therapies in oncology care [22].

Overall, the results showed that many oncologists correctly identified the basic composition of KDs but remained divided on its safety, feasibility, and potential benefit when combined with standard cancer treatments. Key concerns included the risk of malnutrition and patient adherence, especially in young children undergoing intensive therapy. These concerns are justified, as the implementation of the KD requires careful dietary planning, ongoing monitoring, and close coordination between oncologists, dietitians, and caregivers. Similar barriers to recommending dietary therapies have been reported in other contexts, where physicians cite insufficient training, lack of guidelines, and concerns over patient compliance [20,22]. We acknowledge that the use of “perception-positive” and “perception-negative” may imply a dichotomy in correctness. However, given the absence of formal, standardized clinical guidelines for KD use in pediatric oncology, these categories were applied to represent general knowledge levels rather than clinical accuracy. The benchmark used was based on widely accepted scientific literature regarding the KD’s basic macronutrient structure and theoretical mechanisms [10,11], and not on rigid criteria or validated clinical standards.

The multiple correspondence analysis (MCA) further illustrated how perceptions of the KD’s safety, feasibility, and efficacy are interconnected and shaped by factors such as clinical role and training background. Importantly, the study found no strong link between demographic variables and perceptions, suggesting that institutional policies, clinical exposure, and individual experience may play a larger role than gender or years of practice alone.

The MCA results (Figure 1 and Figure 2) illustrate how oncologists’ knowledge and perceptions of KDs are shaped by factors such as professional experience and training background. Physicians with more specialized education or greater experience tended to perceive KDs as somewhat safe and potentially beneficial, whereas less experienced oncologists expressed more concerns about malnutrition risks and side effects. Safety and feasibility emerged as primary determinants of willingness to recommend KDs, often outweighing perceptions of efficacy. Practical barriers—such as patient adherence, cultural dietary preferences, and limited access to specialized dietitians—further influenced attitudes. Addressing these challenges requires a collaborative, multidisciplinary approach with clear, evidence-based guidelines and structured monitoring protocols as described in recent feasibility studies [18,19].

It is essential to emphasize that this was a cross-sectional survey assessing oncologists’ self-reported knowledge and attitudes; it did not collect patient-level clinical or laboratory data. As such, the study does not directly evaluate the clinical efficacy or safety of KD in pediatric patients, nor does it include patient stratification by tumor stage or health status. These limitations have been made explicit to ensure that the findings are interpreted as preliminary and hypothesis-generating. Therefore, the conclusions drawn should be interpreted with caution and not be generalized beyond the context of self-reported perception data. Further studies incorporating longitudinal or mixed-methods designs may provide more robust insight into actual clinical behaviors and patient outcomes.

It is important to acknowledge several limitations of this study. First, the survey was conducted in a single city with a modest sample size, which may limit generalizability to other healthcare settings. Second, the cross-sectional design relied on self-reported perceptions without clinical or laboratory data, so conclusions about the actual safety or efficacy of KDs cannot be drawn. Third, the survey instrument was developed through students’ review and informal pilot testing for face and content validity, but it was not subjected to formal psychometric validation, which may affect the internal consistency, construct validity, and reliability of the findings. Future research should incorporate validated instruments or apply standard psychometric procedures to enhance methodological rigor. Finally, a notable proportion of respondents chose neutral responses on key questions, which may reflect uncertainty, lack of familiarity, or social desirability bias. In addition to knowledge uncertainty, response fatigue or a tendency toward socially desirable answers may have contributed to the high number of neutral selections. These factors are common in self-administered surveys and should be considered when interpreting ambivalent responses.

Future studies should address these limitations by including larger, multi-regions samples, rigorous survey validation, and complementary clinical outcome data. Additionally, most responses came from Center 3, which may introduce sampling bias if perceptions at this center differ from those at smaller sites. However, the exact response rate for each center could not be calculated owing to the unavailability of center-wise data of oncologists. While our survey captured physician-reported concerns about feasibility and supervision, it did not include questions on cost, insurance coverage, or institutional availability of KD services. These are critical factors that may impact KD implementation in real-world settings and should be addressed in future research involving healthcare administrators, dietitians, and policymakers.

This study focused exclusively on oncologists, whose perspectives are shaped by their roles in cancer diagnosis and treatment. However, it would be valuable to compare these findings with insights from nutritionists, endocrinologists, and metabolic specialists, who may have differing levels of familiarity with dietary interventions like the KD. Future interdisciplinary studies could provide a more comprehensive view of barriers and opportunities in KD implementation. Ultimately, high-quality randomized controlled trials and targeted educational initiatives are essential to build oncologists’ confidence and support safe integration of KDs into pediatric oncology care, as emphasized by recent reviews calling for higher-level evidence [17]. However, it is important to recognize that low KD usage may also reflect clinical or infrastructural limitations—such as limited access to specialized dietitians, lack of hospital protocols, or cultural and logistical challenges—rather than knowledge or perception alone. Additionally, this study did not assess perspectives or potential biases from patients’ families or caregivers. Given the central role of parents in pediatric treatment decisions, especially for interventions like KD that require active dietary management at home, future research should include caregiver input to better understand shared decision-making processes and possible social influences on treatment adherence.

Overall, this study underscores the importance of improving oncologists’ knowledge and confidence in discussing emerging dietary approaches such as KDs. By bridging gaps in awareness and developing clear guidelines, the pediatric oncology community can better support families seeking complementary options alongside standard treatments.

## 5. Conclusions

In conclusion, this preliminary study provides insight into oncologists’ knowledge and perceptions of the ketogenic diet (KD) as a potential adjunct therapy for pediatric brain cancer. The findings reveal mixed awareness of the KD’s benefits, with prevailing concerns regarding malnutrition and lack of dietary support, as well as challenges related to adherence. These results suggest a need for further education and exploration of structured guidance, though larger, validated studies are needed to confirm and build upon these findings. As this is an initial, exploratory study based on self-reported survey data from a single region, the findings should be interpreted with caution. Validation of these findings in larger, multicenter studies including diverse patient populations and various tumor types will be crucial to guide the safe and effective integration of KDs into pediatric oncology practice.

## Figures and Tables

**Table 1:** Demographic data of survey participants (*n* = 94).

Factors of Interest	KD Perception		Total	*p*-Value
	Positive (*n* = 47)	Negative (*n* = 47)		
**Gender**				0.535
Female	24 (51.1%)	20 (42.6%)	44 (46.8%)	
Male	23 (48.9%)	27 (57.4%)	50 (53.2%)	
**Professional cadre**				0.313
Consultant	22 (46.8%)	15 (31.9%)	37 (39.4%)	
Assistant consultant	9 (19.1%)	12 (25.5%)	21 (22.3%)	
Fellow	11 (23.4%)	17 (36.2%)	28 (29.8%)	
Resident	5 (10.6%)	3 (6.4%)	8 (8.5%)	
**Professional experience (years)**				0.317
<1	3 (6.4%)	4 (8.5%)	7 (7.4%)	
1–5	17 (36.2%)	16 (34.0%)	33 (35.1%)	
6–10	8 (17.0%)	14 (29.8%)	22 (23.4%)	
10–20	10 (21.3%)	10 (21.3%)	20 (21.3%)	
>20	9 (19.1%)	3 (6.4%)	12 (12.8%)	
**Stream of medical education**				0.922
National only	24 (51.1%)	25 (53.2%)	49 (52.1%)	
International	16 (34.0%)	14 (29.8%)	30 (31.9%)	
International and national	7 (14.9%)	8 (17.0%)	15 (16.0%)	
**Institution**				0.804
Center 1	3 (6.4%)	3 (6.4%)	6 (6.4%)	
Center 2	8 (17.0%)	4 (8.5%)	1 (2.8%)	
Center 3	24 (51.1%)	26 (55.3%)	50 (53.2%)	
Center 4	7 (14.9%)	9 (19.1%)	16 (17.0%)	
Center 5	5 (10.6%)	5 (10.6%)	10.6%)	
**Primary source of KD knowledge**				0.202
Social media	18 (38.3%)	16 (34.0%)	34 (36.2%)	
Medical journals	14 (29.8%)	7 (14.9%)	21 (22.3%)	
Medical school/university	6 (12.8%)	8 (17.0%)	14 (14.9%)	
Family and friends	9 (19.1%)	16 (34.0%)	25 (26.6%)	

**Table 2:** Oncologists’ perception regarding KDs (*n* = 94).

Factors of Interest	KD Perception		Total	*p*-Value
	Positive (*n* = 47)	Negative (*n* = 47)		
**Safety**				0.537
Not safe at all	4 (8.5%)	7 (14.9%)	11 (11.7%)	
Somewhat unsafe	10 (21.3%)	7 (14.9%)	17 (18.1%)	
Neutral	18 (38.3%)	23 (48.9%)	41 (43.6%)	
Somewhat unsafe	13 (27.7%)	8 (17.0%)	21 (22.3%)	
Very safe	2 (4.3%)	2 (4.3%)	4 (4.3%)	
**Ease of use and follow-up**				0.701
Very easy	1 (2.1%)	None	1 (1.1%)	
Easy	6 (12.8%)	4 (8.5%)	10 (10.6%)	
Neutral	9 (19.1%)	13 (27.7%)	22 (23.4%)	
Difficult	25 (53.2%)	26 (55.3%)	51 (54.3%)	
Hard	6 (12.8%)	4 (8.5%)	10 (10.6%)	
**Recent patient queries on KDs**				0.531
Daily	4 (8.5%)	2 (4.3%)	6 (6.4%)	
At least once a week	9 (19.1%)	5 (10.6%)	14 (14.9%)	
At least once a month	7 (14.9%)	12 (25.5%)	19 (20.2%)	
At least once a year	8 (17.0%)	7 (14.9%)	15 (16.0%)	
Never	19 (40.4%)	21 (44.7%)	40 (42.6%)	
**Efficacy of KDs in oncology**				0.536
Agree	21 (44.7%)	25 (53.2%)	46 (48.9%)	
Disagree	26 (55.3%)	22 (46.8%)	48 (51.1%)	
**Willingness to allow KDs**				1.000
Positive	13 (27.7%)	13 (27.7%)	27 (27.7%)	
Negative	12 (25.5%)	13 (27.7%)	25 (26.6%)	
Equivocal	22 (46.8%)	21 (44.7%)	43 (45.7%)	
**Approves KDs for patients**				0.510
Not on active treatment	13 (27.7%)	8 (17.0%)	21 (22.3%)	
On active treatment	12 (25.5%)	14 (29.8%)	26 (27.7%)	
On palliative care	5 (10.6%)	4 (8.5%)	9 (9.6%)	
Off therapy	9 (19.1%)	7 (14.9%)	16 (17.0%)	
None/never	8 (17.0%)	14 (29.85)	22 (23.4%)	

**Table 3:** Use of KDs in practice (*n* = 94).

Factors of Interest	KD Perception		Total	*p*-Value
	Positive (*n* = 47)	Negative (*n* = 47)		
**Reasons for not recommending**				
Supply shortage (+)	13 (27.7%)	17 (36.2%)	30 (31.9%)	0.507
Non-availability of specialized dietitian	20 (42.6%)	22 (46.8%)	42 (44.7%)	0.836
Side effects (dietary)	15 (31.9%)	18 (38.3%)	33 (35.1%)	0.666
Supervision difficulty	24 (51.1%)	21 (44.7%)	45 (47.9%)	0.680
Psychological risk	23 (48.9%)	18 (38.3%)	41 (43.6%)	0.406
Malnutrition/weight loss	26 (55.3%)	29 (61.7%)	55 (58.5%)	0.676
Unknown side effects	26 (55.3%)	16 (34.0%)	42 (44.7%)	0.061
Other(s)	7 (14.9%)	4 (8.5%)	11 (11.7%)	0.523
**If patient follows the KD**				1.000
Will approve to continue	13 (27.7%)	12 (25.5%)	25 (26.6%)	
Disapprove to continue	13 (27.7%)	14 (29.8%)	27 (28.7%)	
Equivocal	21 (44.7%)	21 (44.7%)	42 (44.7%)	

## Data Availability

The original contributions presented in the study are included in the article/Supplementary Materials, further inquiries can be directed to the corresponding author.

## Supplementary Materials

The following supporting information can be downloaded at: https://www.mdpi.com/article/10.3390/nu17172843/s1, Table S1: Summary of MCA; Table S2: Contributions of categorical variables and category levels to dimension 1 in multiple correspondence analysis (MCA); Table S3: Contributions of categorical variables and category levels to dimension 2 in MCA.; Table S4: Contributions of categorical variables and category levels to dimension 3 in MCA.

## Appendix A

Survey Questionnaire

1. Position

- Consultant
- Assistant Consultant
- Fellow
- Resident

2. Gender

- Male
- Female

3. Years of experience

- Under 1 year
- 1 year
- 2 years
- 3 years
- 4 years
- 5 years
- 6 years
- 7 years
- 8 years
- 9 years
- 10 years
- 11 years
- 12 years
- 13 years
- 14 years
- 15 years
- 16 years
- 17 years
- 18 years
- 19 years
- 20 years
- Above 20 years

4. Medical Education

- National (from KSA)
- International (out of KSA)
- National and international

5. Ketogenic diet is:

- Low carbohydrates
- High fat
- High fat and low carbohydrates
- Low protein and carbohydrate
- I don’t know

6. KD is a restricted diet with a uniform regimen for everyone.

- Agree
- Disagree

7. How did you first hear about KDs?

- Social media
- Medical journals
- School/collage
- Family and friends never heard about it

8. Do you think the KD is safe to follow for children with cancer?

- Not safe at all
- Somewhat unsafe
- Neutral
- Somewhat safe
- Very safe

9. Do you believe the KD is easy to follow for children with cancer?

- Very easy
- Easy
- Neutral
- Difficult
- Hard

10. In the past year, how often has an oncology patient or their family members asked you about sugar intake or KDs?

- Daily
- At least once a week
- At least once a month
- At least once a year
- Never

11. The KD is effective in improving treatment outcomes in oncology along with standard of care.

- Agree
- Disagree

12. Are you willing to let your oncology patient follow the KD?

- Yes
- No
- Maybe (depending on the patient’s medical conditions)

13. If you choose maybe, can you explain why?

14. For which of your pediatric cancer patients would you support considering a KD?

Not on treatment

- On radiotherapy/on chemotherapy
- On palliative care
- Off therapy
- None

15. What do you think is the reason(s) behind NOT using the KD in oncology? (Please select all that apply)

- Nutrition supply shortages
- Specialized dietitian
- Dietary side-effect
- Follow-up and supervision
- Other(s)

16. What (if any) are your concerns about recommending a KD? (Choose all that apply)

- No concern
- Risk of psychological harm/anxiety/fear of food
- Risk of weight loss/malnutrition
- Risk of medical side effects
- Other(s)

17. If your patient or their parent told you they follow a KD, what will be your reaction?

- Approve
- Disapprove
- Maybe (depending on patient’s medical conditions)

18. If you choose maybe, can you explain why?
