# Supplementary material for: Oncologists’ Perspectives on Ketogenic Diets in Pediatric Brain Cancer: Potential, Challenges, and the Path Forward
Source: Nutrients. 2025 Aug 31;17(17):2843. doi: 10.3390/nu17172843 (PMC12430085; doi:10.3390/nu17172843)
Supplement: Supplementary file 1 [file nutrients-17-02843-s001.zip › Table S1.pdf]

Table S1. Summary of MCA

Call:

```
MCA(X = Q00, quali.sup = c(2, 3, 4, 5), graph = FALSE)
```

Eigenvalues

	Dim.1	Dim.2	Dim.3	Dim.4	Dim.5	Dim.6	Dim.7	Dim.8	Dim.9	Dim.10
Variance	0.427	0.331	0.310	0.265	0.257	0.231	0.221	0.180	0.169	0.109
% of var.	17.099	13.228	12.405	10.604	10.261	9.250	8.846	7.194	6.767	4.346
Cumulative % of var.	17.099	30.327	42.732	53.336	63.597	72.847	81.693	88.887	95.654	100.000

Individuals (the 20 first)

	Dim.1	ctr	cos2	Dim.2	ctr	cos2	Dim.3	ctr	cos2
1	0.370	0.341	0.134	0.026	0.002	0.001	0.273	0.255	0.073
2	0.274	0.187	0.074	0.202	0.132	0.040	-0.376	0.484	0.138
3	0.737	1.353	0.297	-0.657	1.387	0.235	0.454	0.707	0.112
4	0.948	2.237	0.309	0.675	1.464	0.156	0.498	0.851	0.085
5	-0.734	1.342	0.326	0.761	1.861	0.350	-0.524	0.943	0.166
6	0.274	0.187	0.074	0.202	0.132	0.040	-0.376	0.484	0.138
7	-0.734	1.342	0.326	0.761	1.861	0.350	-0.524	0.943	0.166
8	-0.333	0.275	0.051	0.301	0.292	0.042	-0.300	0.309	0.041
9	0.144	0.052	0.013	-0.544	0.952	0.189	0.458	0.719	0.133
10	-0.865	1.860	0.340	0.014	0.001	0.000	0.309	0.328	0.043
11	-0.813	1.645	0.225	0.558	1.003	0.106	0.273	0.256	0.025
12	-0.350	0.305	0.033	-0.301	0.291	0.024	1.103	4.173	0.325
13	-0.354	0.311	0.120	0.092	0.027	0.008	-0.415	0.590	0.165
14	0.144	0.052	0.013	-0.544	0.952	0.189	0.458	0.719	0.133
15	0.739	1.359	0.211	-0.118	0.045	0.005	-0.991	3.370	0.380
16	0.370	0.341	0.134	0.026	0.002	0.001	0.273	0.255	0.073
17	0.737	1.353	0.297	-0.657	1.387	0.235	0.454	0.707	0.112
18	0.358	0.319	0.040	0.550	0.975	0.095	-1.101	4.158	0.380
19	-0.865	1.860	0.340	0.014	0.001	0.000	0.309	0.328	0.043
20	1.219	3.700	0.399	0.168	0.091	0.008	0.031	0.003	0.000

Categories

	Dim.1	ctr	cos2	v.test	Dim.2	ctr	cos2	v.test	Dim.3	ctr	cos2	v.test
Correct KD Knowledge	-0.125	0.459	0.016	-1.208	0.203	1.555	0.041	1.956	-0.722	21.011	0.521	-6.963
Incorrect KD Knowledge	0.125	0.459	0.016	1.208	-0.203	1.555	0.041	-1.956	0.722	21.011	0.521	6.963
Safety_Neutral	-0.151	0.585	0.018	-1.284	0.802	21.216	0.498	6.804	0.040	0.055	0.001	0.335
Safety_Not safe at all	1.064	7.743	0.150	3.734	0.064	0.036	0.001	0.225	-1.332	16.727	0.235	-4.675
Safety_Somewhat safe	-0.742	7.201	0.159	-3.840	-0.509	4.379	0.075	-2.634	0.452	3.679	0.059	2.338
Safety_Somewhat unsafe	0.809	6.918	0.144	3.665	-0.768	8.073	0.130	-3.482	0.444	2.872	0.043	2.011
Safety_Very safe	-0.913	2.073	0.037	-1.855	-2.459	19.449	0.269	-4.999	-1.003	3.448	0.045	-2.038
Feasibility_Difficult	0.191	1.152	0.043	2.001	-0.664	18.075	0.523	-6.972	-0.197	1.695	0.046	-2.067
Feasibility_Easy	-1.011	6.359	0.122	-3.364	0.409	1.346	0.020	1.361	1.335	15.293	0.212	4.443
Feasibility_Hard	1.702	18.022	0.345	5.663	0.828	5.513	0.082	2.755	0.305	0.799	0.011	1.016
Feasibility_Neutral	-0.806	8.883	0.198	-4.294	0.875	13.537	0.234	4.663	-0.441	3.676	0.060	-2.353
Feasibility_Very easy	1.095	0.746	0.013	1.095	2.242	4.043	0.054	2.242	3.343	9.583	0.120	3.343
Efficacious	-0.838	20.119	0.674	-7.915	-0.130	0.624	0.016	-1.226	-0.044	0.077	0.002	-0.418
Non-Efficacious	0.804	19.281	0.674	7.915	0.124	0.598	0.016	1.226	0.042	0.074	0.002	0.418

Categorical variables (eta2)

	Dim.1	Dim.2	Dim.3
Perception	0.016	0.041	0.521
Safety	0.419	0.703	0.332
Feasibility	0.601	0.562	0.385
Efficacy	0.674	0.016	0.002

Supplementary categories

	Dim.1	cos2	v.test	Dim.2	cos2	v.test	Dim.3	cos2	v.test
Assistant Consultant	-0.222	0.014	-1.146	-0.083	0.002	-0.427	-0.227	0.015	-1.172
Consultant	0.029	0.001	0.226	-0.063	0.003	-0.489	0.000	0.000	-0.003
Fellow	0.289	0.035	1.813	0.044	0.001	0.279	0.012	0.000	0.078
Resident	-0.563	0.029	-1.656	0.352	0.012	1.036	0.553	0.028	1.627
Female	-0.062	0.003	-0.565	-0.031	0.001	-0.281	0.167	0.024	1.507
Male	0.055	0.003	0.565	0.027	0.001	0.281	-0.147	0.024	-1.507
1 to <=5 year	-0.035	0.001	-0.249	0.015	0.000	0.106	0.070	0.003	0.497
10 to <=20 year	0.247	0.016	1.237	-0.171	0.008	-0.859	-0.241	0.016	-1.206
6 to <=10 year	-0.031	0.000	-0.164	0.148	0.007	0.787	-0.049	0.001	-0.261
Above 20 year	-0.123	0.002	-0.454	-0.092	0.001	-0.338	0.329	0.016	1.213
Less thsn 1 year	-0.232	0.004	-0.635	0.112	0.001	0.306	-0.053	0.000	-0.144
International out of KSA	-0.035	0.001	-0.228	-0.316	0.047	-2.086	-0.019	0.000	-0.123
National from KSA	-0.038	0.002	-0.383	0.137	0.020	1.375	0.041	0.002	0.414
National and International	0.193	0.007	0.812	0.185	0.007	0.779	-0.097	0.002	-0.408

Supplementary categorical variables (eta2)

	Dim.1	Dim.2	Dim.3
Position	0.063	0.014	0.038
Gender	0.003	0.001	0.024
Experience	0.020	0.013	0.029
Med.Education	0.007	0.047	0.002
