# Supplementary material for: Oncologists’ Perspectives on Ketogenic Diets in Pediatric Brain Cancer: Potential, Challenges, and the Path Forward
Source: Nutrients. 2025 Aug 31;17(17):2843. doi: 10.3390/nu17172843 (PMC12430085; doi:10.3390/nu17172843)
Supplement: Supplementary file 1 [file nutrients-17-02843-s001.zip › Table S2.pdf]

Table S2. Contributions of Categorical Variables and Category Levels to Dimension 1 in Multiple Correspondence Analysis (MCA)

Categorical variables	R^2	Estimate	p.value
**Efficacy**	**6.737E-01**		**4.257E-24**
Efficacy=non-efficacious		5.368E-01	4.257E-24
Efficacy=Efficacious		-5.368E-01	4.257E-24
**Feasibility**	**6.013E-01**		**4.724E-17**
Feasibility=Feasibility Hard		9.597E-01	4.936E-10
Feasibility=Feasibility Difficult		-2.853E-02	4.474E-02
Feasibility=Feasibility Easy		-8.141E-01	5.696E-04
Feasibility=Feasibility Neutral		6.798E-01	6.878E-06
**Safety**	**4.192E-01**		**6.182E-10**
Safety=Safety Not safe at all		6.868E-01	1.155E-04
Safety=Safety Somewhat unsafe		5.201E-01	1.583E-04
Safety=Safety Somewhat safe		-4.940E-01	7.050E-05
