# Supplementary material for: Oncologists’ Perspectives on Ketogenic Diets in Pediatric Brain Cancer: Potential, Challenges, and the Path Forward
Source: Nutrients. 2025 Aug 31;17(17):2843. doi: 10.3390/nu17172843 (PMC12430085; doi:10.3390/nu17172843)
Supplement: Supplementary file 1 [file nutrients-17-02843-s001.zip › Table S3.pdf]

Table S3. Contributions of Categorical Variables and Category Levels to Dimension 2 in MCA.

Categorical variables	R^2	Estimate	p.value
**Safety**	**7.031E-01**		**1.094E-22**
Safety=Safety Neutral		7.914E-01	2.034E-15
Safety=Safety Somewhat safe		3.729E-02	7.736E-03
Safety=Safety Somewhat unsafe		-1.118E-01	3.499E-04
Safety=Safety Very safe		-1.084E+00	8.722E-08
**Feasibility**	**5.624E-01**		**2.782E-15**
Feasibility=Feasibility Neutral		7.861E-02	7.956E-07
Feasibility=Feasibility Hard		5.172E-02	5.255E-03
Feasibility=Feasibility Very easy		8.650E-01	2.414E-02
Feasibility=Feasibility Difficult		-8.062E-01	1.908E-16
**Perception**	**4.115E-02**		**4.990E-02**
Perception=No knowledge on KD		1.167E-01	4.990E-02
Perception=Positive knowledge on KD		-1.167E-01	4.990E-02
