# Supplementary material for: Oncologists’ Perspectives on Ketogenic Diets in Pediatric Brain Cancer: Potential, Challenges, and the Path Forward
Source: Nutrients. 2025 Aug 31;17(17):2843. doi: 10.3390/nu17172843 (PMC12430085; doi:10.3390/nu17172843)
Supplement: Supplementary file 1 [file nutrients-17-02843-s001.zip › Table S4.pdf]

Table S4. Contributions of Categorical Variables and Category Levels to Dimension 3 in MCA.

Categorical variables	R^2	Estimate	p.value
**Perception**	**5.213E-01**		**2.189E-16**
Perception=Knowledge on KD		4.021E-01	2.189E-16
Perception=No knowledge on KD		-4.021E-01	2.189E-16
**Feasibility**	**3.851E-01**		**7.238E-09**
Feasibility=Feasibility Easy		2.597E-01	2.967E-06
Feasibility=Feasibility Very easy		1.378E+00	6.204E-04
Feasibility=Feasibility Difficult		-5.936E-01	3.799E-02
Feasibility=Feasibility Neutral		-7.298E-01	1.780E-02
**Safety**	**3.322E-01**		**2.479E-07**
Safety=Safety Somewhat safe		4.075E-01	1.856E-02
Safety=Safety Somewhat unsafe		4.030E-01	4.367E-02
Safety=Safety Very safe		-4.025E-01	4.085E-02
Safety=Safety Not safe at all		-5.858E-01	7.377E-07
